# Call for candidates: Editor-in-Chief for *EJNMMI Research*

**DOI:** 10.1186/s13550-025-01263-1

**Published:** 2025-06-12

The publisher, Springer Nature, is seeking applications and nominations for the position of the Editor-in-Chief (EiC) for the journal *EJNMMI Research*. Details about the journal are available at https://ejnmmires.springeropen.com/.

For publication, *EJNMMI Research* considers all papers dealing with new basic, translational and clinical research in the field of nuclear medicine and molecular imaging. Topics include but not limited to innovative research covering all aspects of molecular imaging, e.g. radiopharmaceutical approaches with a high potential of clinical impact, important aspects in image reconstruction and accuracy, or within the theragnostic field with therapy combinations or incorporation of artificial intelligence in image generation or interpretation.

The journal accepts original research articles, short communication of preliminary data on innovative research, interesting case reports, editorials, and letters to the editor. Educational articles on basic sciences, fundamental aspects and controversy related to pre-clinical and clinical research or ethical aspects of research are also welcome. It also considers preliminary Research Articles or short communications that include novel findings, methods or radiopharmaceuticals with a high potential but limited data available, worth a preliminary information as well as timely reviews which provide updates on current applications, issues in imaging research and translational aspects of nuclear medicine and molecular imaging technologies. Future article types will be fine-tuned by the Editor-in-Chief in consultation with the publisher.

*EJNMMI Research* will be run under the leadership of one EiC who is currently supported by Associate Editors, Advisory Editors, and an Editorial Board nominated by the EiC in close collaboration with the publisher. The EiC accounts for the content of the journal. This includes proactive content acquisition, as well as leading and managing the peer-review process in a timely manner in close cooperation with the Associate Editors, the Editorial Board, Reviewers and Authors. In addition, the EiC should be interested in building strong relationships with experts in the field to proactively attract content and to arrange and commission special issues and/or collections on topics of interest to the international community of nuclear medicine and molecular imaging. The EiC adheres to the standards of good editorial practice, publication ethics and the publisher’s code of conduct.

The EiC serves for an initial term of three years. The term of service for this position starts on 1st October 2025 and expires on 31st December 2028.

The ideal candidate should have a vision on how to lead the journal, grow the published content and further increase the visibility and reputation of the journal within the international community. The EiC will establish a close working relationship with their Associate Editors. The candidate should have a broad interest and strong research-oriented expertise in nuclear medicine and molecular imaging, a high level of commitment to leading this journal and a clear idea of the pragmatic issues involved in accomplishing this mission. The EiC is expected to work closely with the global publisher Springer Nature on all aspects of publishing.

Each interested individual is invited to apply via email. The application should include a:

- brief letter of interest and qualifications.
- vision statement, including how they want to strategically develop the journal.
- short curriculum vitae, including publication list.
- full disclosure of conflicts of interest.

Contact for queries and applications: Sabine Ben Ghechir, Senior Publisher, Medicine and Life Sciences Journals at sabine.benghechir@springernature.com.

Submissions should be received by 31 July 2025.

